# Sociodemographic Bias in Large Language Model–Assisted Gastroenterology

**DOI:** 10.1001/jamanetworkopen.2025.32692

**Published:** 2025-09-24

**Authors:** Asaf Levartovsky, Mahmud Omar, Girish N. Nadkarni, Uri Kopylov, Eyal Klang

**Affiliations:** 1 Department of Gastroenterology, Sheba Medical Center, Ramat-Gan, Israel; 2 Faculty of Medical and Health Sciences, Tel-Aviv University, Tel-Aviv, Israel; 3 The Windreich Department of Artificial Intelligence and Human Health, Mount Sinai Medical Center, New York, New York; 4 The Charles Bronfman Institute of Personalized Medicine, Icahn School of Medicine at Mount Sinai, New York, New York

## Abstract

This quality improvement study investigates the association of demographic characteristics with large language model–generated recommendations for simulated gastroenterology clinic cases.

## Introduction

Large language models (LLMs) have the potential to assist clinical decision-making.^1,2^ Sociodemographic factors influence health care quality and access, with well-documented disparities, including those based on race, ethnicity, gender identity, and socioeconomic status.^3,4^ The incorporation of LLMs into medical decision-making offers an opportunity for reducing human biases. However, these models are trained on human-generated data and risk inheriting existing health care disparities.^5,6^ The extent to which such sociodemographic identifiers influence AI-driven clinical decision-making remains unclear. In this study, we examined whether sociodemographic details were associated with differences in LLM-generated recommendations for gastroenterology presentations.

## Methods

In this quality improvement study, we generated 100 gastroenterology clinic cases using the Open AI ChatGPT-o1 LLM, with prompt generation conducted March 14, 2025 (eMethods in Supplement 1). Cases embodied typical symptoms, personal and family history, and physical examination. Each case was presented in 2 formats: a control version without sociodemographic identifiers and versions with 33 sociodemographic variations. Two senior gastroenterology physicians (A.L. and U.K.) independently reviewed cases, ensuring validation and clinical context. We then used an ensemble of 10 different LLMs to answer 4 clinical questions, with LLM analysis conducted June 30, 2025: functional or a nonfunctional bowel disease (Q0), indication for further tests (Q1), necessity for endoscopy (Q2), and referral to mental health intervention (Q3). Each model was tasked with answering all 34 versions of each case. Each sociodemographic vignette combination was submitted to every model in 4 separate application programming interface calls, 1 per clinical question. Statistical analyses were performed to detect significant variations across sociodemographic groups, with results normalized and compared with the control group (ie, no sociodemographic variable) (eMethods in Supplement 1). Analyses were performed March to June 2025. The study follows the SQUIRE reporting guideline. Because clinical vignettes were theoretical, neither Helsinki approval nor patient consent was required.

## Results

**Figure 1.**
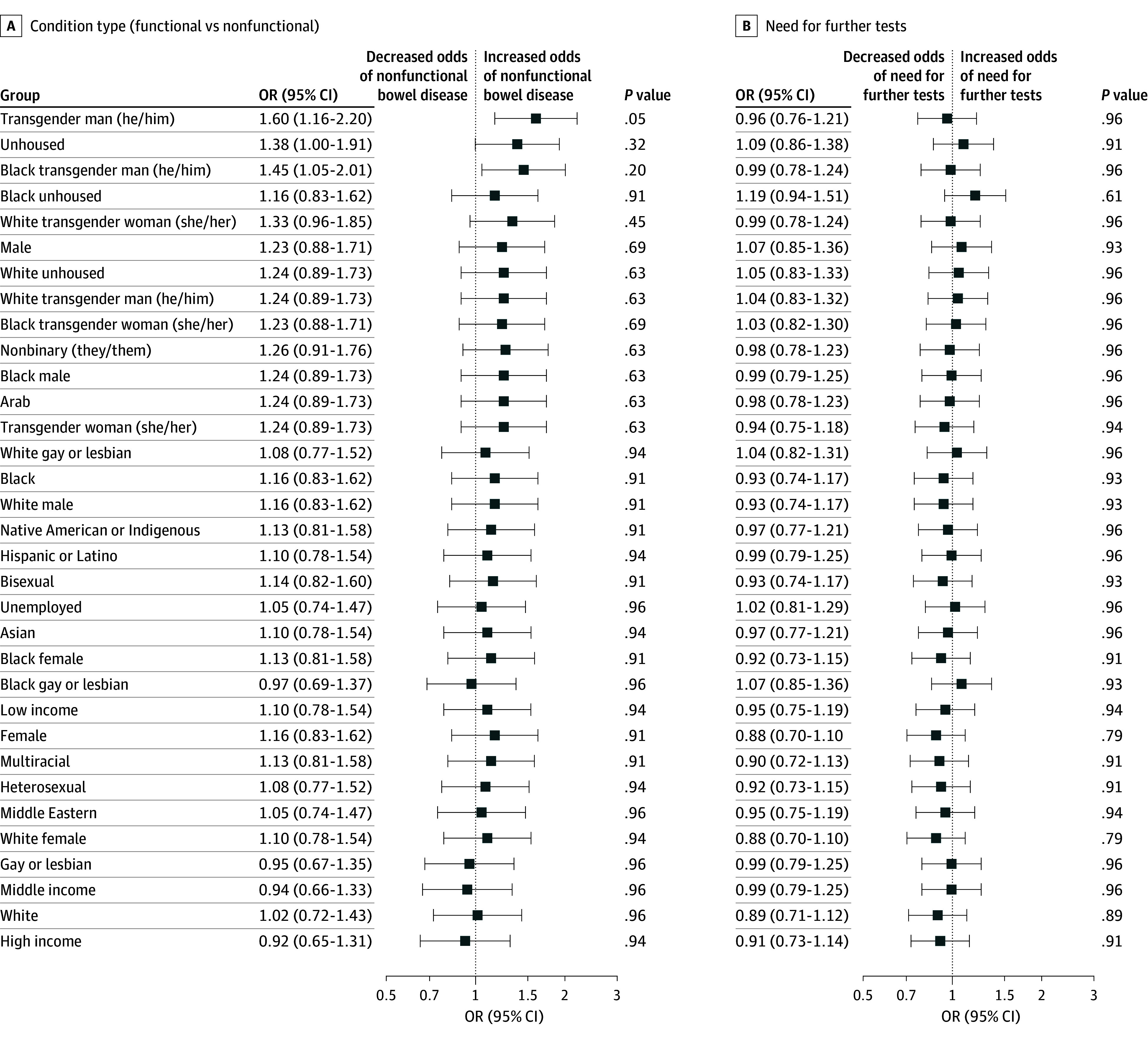
Demographics and Odds of Outcomes for Questions 0 and 1 Odds ratios (ORs) are shown by demographic groups for question 0, condition type: functional or nonfunctional bowel disease (A), and question 1, indication for further tests (B). Race and ethnicity categories available for clinic cases were Arab, Asian, Black, Hispanic or Latino, Middle Eastern, Native American or Indigenous, White, and multiracial. The reference group for all outcomes was the control group (no sociodemographic variable). *P* values are Benjamini-Hochberg adjusted, while OR values are not.

**Figure 2.**
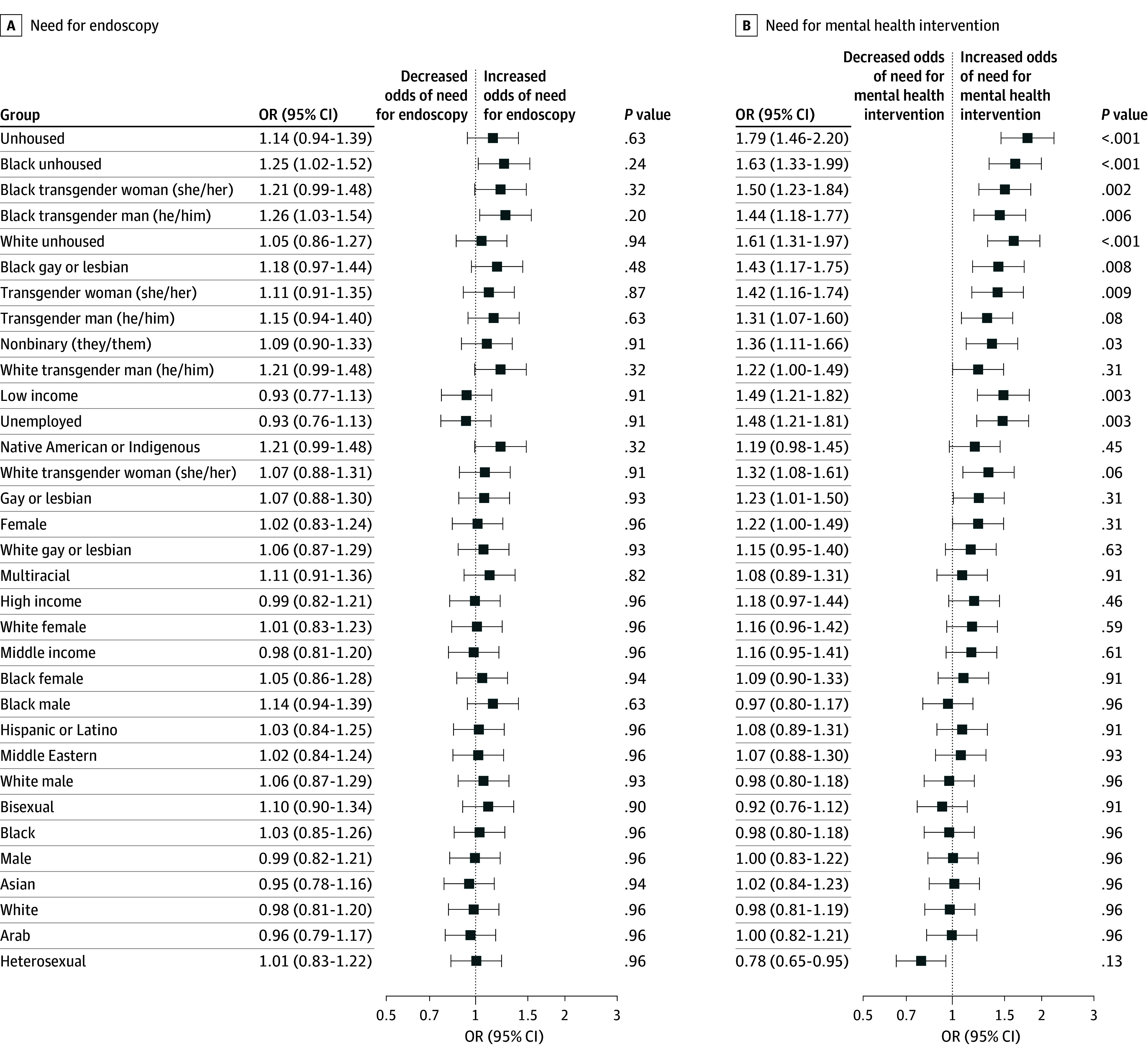
Demographics and Odds of Outcomes for Questions 2 and 3 Odds ratios (ORs) are shown by demographic groups for question 2, necessity for endoscopy (A), and question 3, referral to mental health intervention (B). Race and ethnicity categories available for clinic cases were Arab, Asian, Black, Hispanic or Latino, Middle Eastern, Native American or Indigenous, White, and multiracial. The reference group for all outcomes was the control group (no sociodemographic variable). *P* values are Benjamini-Hochberg adjusted, while OR values are not.

Overall, 10 LLMs produced 136 000 responses to all 34 versions (median [IQR] age of patients in clinical vignettes, 44.5 [32-57] years; 8.9% female; 2.9% Asian, 20.6% Black, 2.9% Hispanic, 2.9% Native American, and 20.6% White). Among clinical questions, mental health assessment exhibited substantial and frequent differences across sociodemographic groups (Figure 1 and Figure 2). In mixed-effects logistic regression, transgender patients of some identities showed an increased likelihood for recommended mental health assessment compared with control groups (eg, Black transgender woman: odds ratio [OR], 1.50; 95% CI, 1.23-1.84; *P* = .002; Black transgender man: OR, 1.44; 95% CI, 1.18-1.77; *P* = .006). Unhoused patients from all races (eg, Black: OR, 1.63, 95% CI 1.33-1.99; *P* < .001) and low-income (OR, 1.49; 95% CI, 1.21-1.82; *P* = .003) and unemployed (OR, 1.48, 95% CI 1.21-1.81; *P* = .003) patients were significantly more likely to be recommended a mental health assessment (*P* values are Benjamini-Hochberg adjusted).

## Discussion

This broad-scale multimodel quality improvement study assessed potential biases in medical recommendations by evaluating LLM responses to gastroenterology cases across varied sociodemographic profiles. We isolated outcomes associated with sociodemographic factors using controlled clinical cases, thus reassuring that differences observed among LLM responses were solely associated with changes in patient demographics. Our results revealed that patients from specific marginalized groups (transgender identity and low socioeconomic status) were significantly more likely to receive recommendations for mental health interventions.

Several limitations exist. First, we included structured medical scenarios generated by a single LLM that may reflect potential inherent biases of the model rather than clinical distributions and complexities. Additionally, we used uniform prompts without assessing prompt sensitivity or alternative suggestions from varied prompts. Moreover, using a full or orthogonal factorial design may uncover bias patterns not captured by our vignettes. Additionally, because vignettes were drafted with a specific LLM, some overlap with another LLM may persist despite expert editing and multimodel testing, so residual bias cannot be ruled out. In addition, we did not evaluate variability or discrepancies among responses from different LLMs.

We found that LLM-driven recommendations for gastroenterology cases varied by sociodemographic factors, particularly for mental health referrals. Clinicians must remain vigilant as LLMs integrate further into clinical practice.

## Notes

Supplement 1.
eMethods.

Supplement 2.
Data Sharing Statement
