# Supplementary material for: Sociodemographic Bias in Large Language Model–Assisted Gastroenterology
Source: JAMA Netw Open. 2025 Sep 24;8(9):e2532692. doi: 10.1001/jamanetworkopen.2025.32692 (PMC12461430; doi:10.1001/jamanetworkopen.2025.32692)
Supplement: Supplement 2. — Data Sharing Statement [file jamanetwopen-e2532692-s002.pdf]

**Data Sharing Statement**

Levartovsky. Sociodemographic Bias in Large Language Model–Assisted Gastroenterology. *JAMA Netw Open*. Published online September 24, 2025. doi:10.1001/jamanetworkopen.2025.32692

**Data**
**Data available:** Yes
**Data types:** Data (not involving human participants)
**How to access data:** If data is a requisition, I'll provide access to it. **When available:** With publication

**Supporting Documents**
**Document types:** None

**Additional Information**
**Who can access the data:** researchers whose proposed use of the data has been approved
**Types of analyses:** for any purpose or for a specified purpose
**Mechanisms of data availability:** after approval of a proposal
